# HPLC with charged aerosol detector (CAD) as a quality control platform for analysis of carbohydrate polymers

**DOI:** 10.1186/s13104-019-4296-y

**Published:** 2019-05-14

**Authors:** Rajarshi Ghosh, Paul Kline

**Affiliations:** Department of Chemistry, Middle Tennessee State University, 1301 E Main Street, MTSU Box 68, Murfreesboro, TN 37132 USA

**Keywords:** HILIC, HPSEC, QC of carbohydrates, Hyaluronic acid, Uronic acids, Amino sugars

## Abstract

**Objective:**

QC analysis of carbohydrates has been historically cumbersome due to lengthy and laborious derivatization techniques and the requirement of complimentary instrumentation. HILIC-CAD has emerged as an effective platform for direct monosaccharide composition analysis of complex carbohydrates without derivatization. Although, several neutral sugars have been separated and detected using HILIC-CAD, there has not been any report on acidic and amino sugar analysis using this method. In this study, we developed a gradient method for simultaneous analysis of acidic, amino and select neutral monosaccharides. As an application of the HILIC-CAD method, we performed composition analysis of commercially purchased hyaluronic acid products. Additionally, since CAD is suitable for SEC experiments, we tested the homogeneity of hyaluronic acids using a SEC-CAD method.

**Results:**

We separated common uronic acids (GlcA, GalA, LIdoA and Neu5Ac), amino sugars (GlcN, GalN and GlcNAc) and select neutral sugars (LRha, LFuc, Man and Gal) using a gradient HILIC-CAD method. The optimized gradient method demonstrated good linearity (R^2^ > 0.99), precision (RSD < 8%), LOD (< 85 ng/mL) and LOQ (< 280 ng/mL). HILIC-CAD analysis of commercially purchased hyaluronic acid products indicated that samples were composed of GlcNAc and GlcA. Additionally, SEC-CAD chromatograms indicated the heterogeneous nature of the samples.

**Electronic supplementary material:**

The online version of this article (10.1186/s13104-019-4296-y) contains supplementary material, which is available to authorized users.

## Introduction

Carbohydrates have become increasingly important commercially not only as food or structural building blocks, but also as natural health products [1]. It is essential to have proper QC of carbohydrate-based therapeutics to ensure their efficacy and safety. Physicochemical properties such as homogeneity, size and composition serve as important QC parameters for carbohydrates [1]. The QC process is often time-consuming and expensive due to the lack of a single platform capable of analyzing necessary parameters. Analysts rely on laborious derivatization procedures and complementary analytical instrumentation to test products. These problems underline the need for a simple multipurpose platform for rapid QC of carbohydrates.

Analysis of complex carbohydrates has been historically challenging due to their heterogeneity and diversity [1]. Additionally, monosaccharide composition analysis can be tricky due to the presence of epimers, formation of anomers and lack of a chromophore [2]. Common analytical techniques (GC–MS and reverse phase HPLC) for composition analysis require appropriate derivatization of monosaccharides [3, 4]. As a result analysis of underivatized monosaccharides is becoming increasingly popular. High performance anion exchange chromatography with pulsed amphoteric detection (HPAEC-PAD) has proven to be an effective tool for direct analysis of monosaccharides in recent years [5]. However, the high pH of the eluent resulting in epimerization and degradation of carbohydrates, unstable baseline, loss of sensitivity and requirement of a dedicated base-compatible HPLC are some of its disadvantages [6]. Hydrophilic interaction liquid chromatography (HILIC) coupled to evaporative light scattering detector (ELSD), mass spectrometer (MS), refractive index detector (RID) and charged aerosol detector (CAD) offer other alternate ways of analyzing underivatized carbohydrates [2, 7–10]. The CAD, introduced by Dixon and Peterson [11], offers several advantages compared to other detectors used in the direct analysis of sugars. The response of CAD does not depend on the structural properties of the analyte and it offers greater sensitivity than ELSD [12]. It is compatible with gradient elution and allows detection of all non-volatile and most semi-volatile analytes [13]. It is also relatively cheap and easy to use compared to MS. Hence, HILIC coupled with CAD can be an excellent tool for direct composition analysis and detection of impurities in samples. Although, neutral sugars have been previously separated using HILIC-CAD, there has not been any reports on the analysis of acidic and amino sugars.

In this study, a method has been developed to separate and detect amino sugars (GlcN, GalN and GlcNAc) and acidic sugars (GlcA, GalA, Neu5Ac and LIdoA) without derivatization using HILIC-CAD. Commonly found N-linked neutral sugar residues in mammalian glycoproteins such as LFuc, Gal and Man were also simultaneously separated. As an application of our proposed QC platform, we analyzed the monomer composition of commercially available hyaluronic acid (HA) products.

CAD has also been shown to be effective in size exclusion chromatography (SEC) with better impurity and polydispersity profiles compared to RID ad ELSD [14]. Therefore, SEC-CAD can be potentially employed for homogeneity and molecular weight analysis of carbohydrates. In this study, we demonstrated the homogeneity of commercially purchased HA products using a SEC-CAD method adapted from Chen et al. [15].

## Main text

### Materials and methods

#### Material and reagents

All standards, solvents and buffer additives were of HPLC grade (Sigma Aldrich, Synthose Inc., and Fisher Scientific). HA supplements were purchased from local supermarkets.

#### Separation of monosaccharides by HILIC-CAD

A Dionex Ultimate 3000 HPLC system coupled to a Corona charged aerosol detector was used for the chromatographic analysis. Separation was carried out using a Waters XBridge BEH Amide XP (3 × 150 mm; 2.5 µm) column. The mobile phase of the optimized method consisted of (A) 90% acetonitrile with 0.2% TEA and 25 mM ammonium acetate; and (B) water with 0.2% TEA and 25 mM ammonium acetate. The following gradient elution was used: 0% B at 0–15 min, 0–18% B at 15–40 min, 18% B at 40–45 min, 18–0% B at 45–47 min and 0% B at 47–55 min. The flow rate was 0.5 mL/min. A column temperature of 50 °C and an injection volume of 10 µL (mixed standard monosaccharide solution at a concentration of 225 µg/mL) was used for the analysis. The effects of column temperature (50 °C, 40 °C and 30 °C) and buffers such as ammonium acetate (25 mM and 20 mM), ammonium formate (25 mM and 20 mM) and triethylamine (TEA) (0.2% and 0.1%) on chromatographic separation were evaluated. The following parameters were used for the CAD detector: (i) nitrogen gas pressure: 35 psi; (ii) detector response 100 pA; and (iii) noise filter: high. Data processing was carried out using Chromeleon 6.8 software.

#### Validation

Seven different concentrations (15–1000 µg/mL) of the standards were injected in triplicate to construct a double logarithmic plot, which was used as a calibration curve [16]. The limit of detection (LOD) and limit of quantification (LOQ) was calculated based on previously reported methods [16, 17]. Intra-day precision (%RSD) was determined by repeating the analysis of a standard solution five times in a single day. Inter-day precision (%RSD) analysis was performed over 3 days.

#### Application of HPLC-CAD as a QC platform

The monosaccharide composition of commercially purchased HA products were analyzed to demonstrate the application of the HILIC-CAD method. Samples (> 5 mg) were hydrolyzed using 2 mL of 2 M trifluoroacetic acid (TFA) at 110 °C for 2 h prior to composition analysis.

The homogeneity of HA was also analyzed by a separate high performance size exclusion chromatography (HPSEC) experiment. GPC grade HA standards and commercially purchased samples were analyzed on a TOSOH TSKgel G4000PW_xl_ (7.8 × 300 mm; 10 µm) column for 35 min. Ammonium acetate (25 mM) at a flow rate of 0.5 mL/min was used as the eluent. A volume of 10 µL of sample was injected into the column. The CAD parameters were the same as before.

### Results and discussion

#### Separation of monosaccharides by HILIC-CAD

**Fig. 1:**
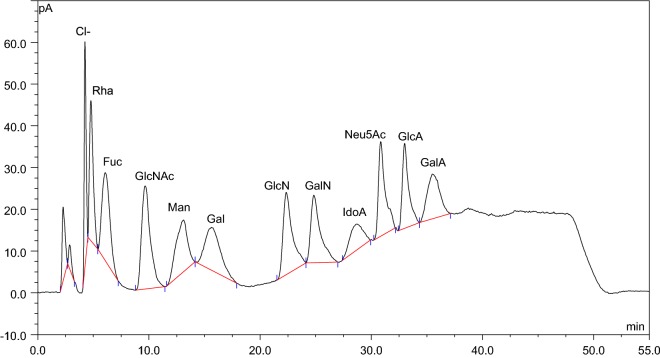
Separation of monosaccharides by HILIC-CAD. The HILIC-CAD gradient method was able to separate a mixture of 11 monosaccharide standards (225 µg/mL) (Rha, Fuc, GlcNAc, Man, Gal, GlcN, GalN, IdoA, Neu5Ac, GlcA and GalA) in this study. Buffer additives of 25 mM ammonium acetate and 0.2% TEA and a column temperature of 50 °C were used for the HPLC analysis

The gradient method was able to separate uronic acids (GlcA, GalA, Neu5Ac and LIdoA) and amino sugars (GlcN, GalN and GlcNAc) as well as select neutral sugar residues (LRha, LFuc, Man and Gal) (Fig. 1). The deoxy monosaccharides eluted first followed by acetylated amino sugars, aldohexoses, amino sugars and uronic acids. This method can be potentially adapted for the monosaccharide composition analysis of other acidic/amino sugar-rich polysaccharides such as glycosaminoglycans and N-linked sugar residues found in mammalian glycoproteins.

A major problem in analyzing monosaccharides is the formation of α and β anomers resulting in split peaks. Addition of tertiary amines in the mobile phase, high temperature and high pH are common ways to increase the rate of the interconversion of anomers [9, 18]. During method development, different column temperatures (30 °C, 40 °C and 50 °C) (Additional file 1: Fig. S1) and concentrations of TEA in the mobile phase (0.1% and 0.2%) (Additional file 1: Fig. S2) were used to suppress split peaks. Although, temperatures below 30 °C were able to separate most monosaccharides, it produced anomeric peak doublets (data not shown). Column temperature of 50 °C was able to adequately separate acidic and amino sugars along with select neutral sugars without formation of split peaks (Fig. 1). Addition of TEA in the mobile phase was also found to enhance mutarotation rates to yield single peaks (Additional file 1: Fig. S2). A concentration of 0.1% TEA was found to be sufficient to suppress peak doublets for most monosaccharides except mannose (Additional file 1: Fig. S2). Hence, a combination of 0.2% TEA and 50 °C column temperature was chosen for the optimized method. Higher concentrations of TEA (> 0.2%) or high pH (> 10) caused substantial detector noise and unstable baseline. Apart from tertiary amines, we also tested the effects of ammonium acetate (Additional file 1: Fig. S3) and ammonium formate (data not shown), on separation. Ammonium formate gave a slightly higher detector response compared to ammonium acetate (data not shown). However, ammonium acetate gave a more stable baseline and was subsequently chosen as the buffer of choice. Although, higher ammonium acetate concentrations increased retention times, it also resulted in better separations (Additional file 1: Fig. S3). Ammonium acetate concentrations above 25 mM led to high detector noise.

The optimized method was validated by evaluating linearity, LOD, LOQ and intra and inter-day precision of peak areas (Additional file 1: Table S1). After double logarithmic transformation, the calibration curves of 11 analytes showed good linearity (> 0.99). The LOD and LOQ values were in the range of 50–83 ng/mL and 170–278 ng/mL respectively, which were higher compared to earlier studies involving neutral sugars [9, 16]. The higher LOD and LOQs reported in this study might be due to the higher concentrations of ammonium acetate (25 mM) and TEA (0.2%) in the mobile phase. The results indicated satisfactory intra and inter-day precision of peak areas (1–8% RSD).

#### HPLC-CAD as a QC platform

**Fig. 2:**
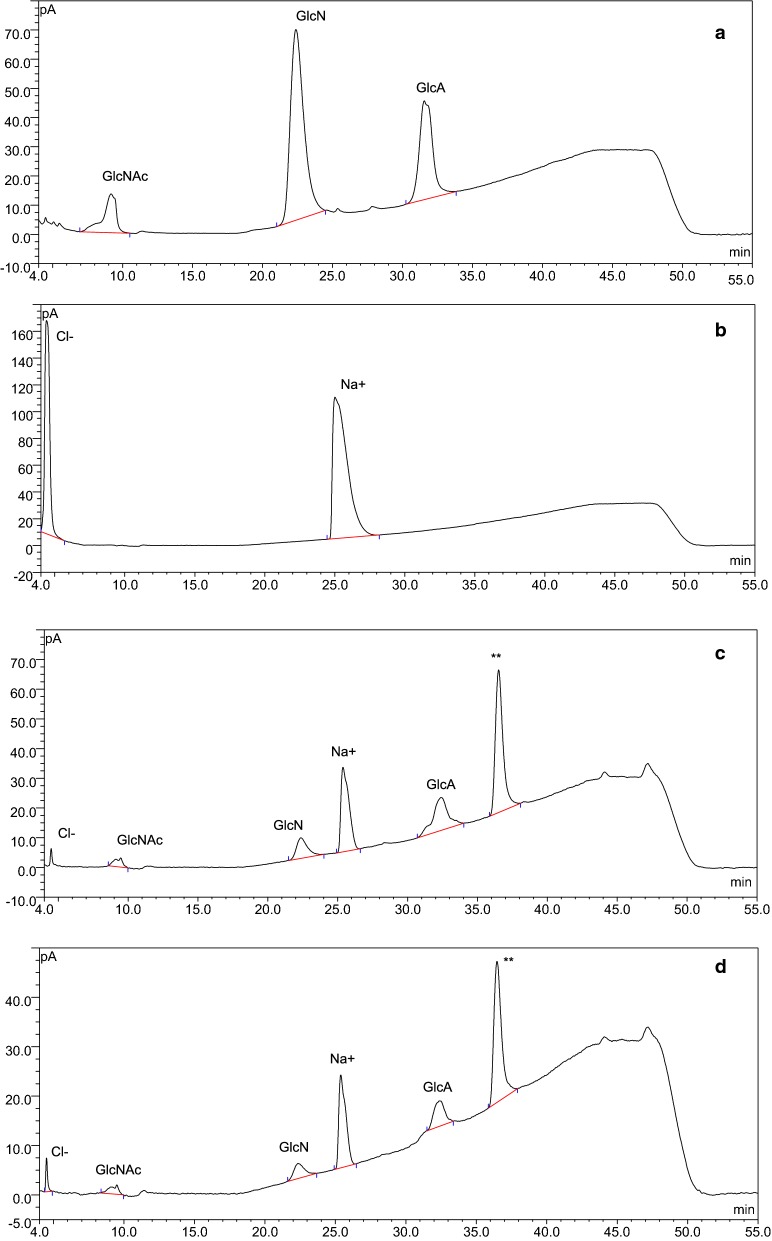
Monosaccharide composition of HA samples by HILIC-CAD. Authentic standards and commercially purchased HA samples were hydrolyzed by 2 M TFA for 2 h before composition analysis by the HILIC-CAD gradient method. **a** Represents a chromatogram of GlcNAc and GlcA standards. The degradation of GlcNAc into GlcN is evident from the chromatogram. **b** Is a representative chromatogram of NaCl solution. The sodium and chloride ion peaks are visible in the chromatogram. **c** Shows a chromatogram of a hydrolyzed HA standard. The Cl^−^, GlcNAc, GlcN, Na^+^ and GlcA peaks can be seen along with an unknown peak (**). The unknown peak is speculated to be a disaccharide repeating unit of HA. **d** Is a representative chromatogram of a hydrolyzed HA serum sample. The composition of **d** is identical to **c** indicating that it is an authentic product

The glycosaminoglycan HA, a widely used dietary supplement, was used to demonstrate the utility of HPLC-CAD as a QC platform. HA is composed of a repeating disaccharide unit made up of GlcNAc and GlcA [19]. The HILIC-CAD gradient method was used to analyze the composition of a HA standard and a commercially available HA product (Fig. 2). Figure 2a shows the chromatogram of GlcNAc and GlcA standards treated with 2 M TFA at 110 °C for 2 h. The breakdown of GlcNAc into GlcN is evident from the chromatogram. Figure 2b represents a chromatogram of NaCl solution treated under the same conditions as in Fig. 2a. The HA standard used in our study was a sodium salt. Since CAD is a quasi-universal detector, it is likely that sodium and chloride ion peaks would show up in the chromatograms. The respective sodium and chloride ion peaks are visible in Fig. 2b. Figure 2c shows the chromatogram of an acid hydrolyzed HA standard. The chromatogram indicates the presence of GlcN, GlcA, sodium, chloride and minor amounts of GlcNAc. The unknown peak is likely to be a disaccharide unit of GlcNAc and GlcA. Figure 2d shows the chromatogram of an acid hydrolyzed HA serum sample. The contents of the sample in Fig. 2d are identical to the HA standard indicating its authenticity. This HILIC-CAD method is easily adaptable to other similar carbohydrate-based therapeutics. The method is easy to execute and saves time compared to other laborious techniques involving derivatization. The entire monosaccharide composition analysis (hydrolysis of samples and HPLC analysis) using our method can be completed within 3 h. This is a significant improvement compared to other traditional methods involving derivatization which can take > 24 h.

**Fig. 3:**
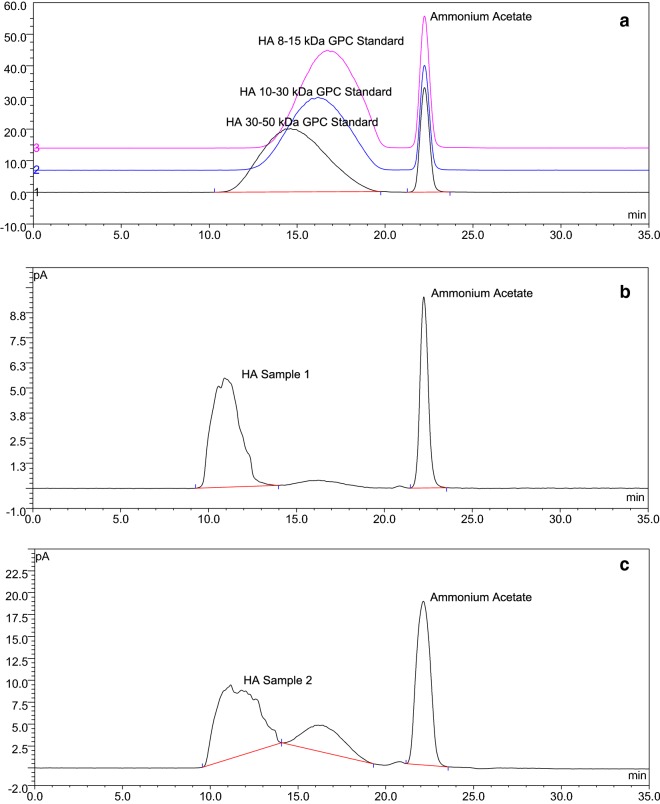
Purity and homogeneity analysis of HA samples by HPSEC-CAD. **a** Shows the overlay of HPSEC chromatograms of HA GPC standards of varying molecular weights (pink: 8–15 kDa; blue: 10–30 kDa; black: 30–50 kDa). **b**, **c** Represent HPSEC chromatograms of commercially available HA products. Although **b** is predominantly composed of a single peak, the shape of the peak indicates that it might not be a homogeneous fraction. **c** Contains two non-symmetrical peaks indicating that it is a heterogeneous sample

The homogeneity of HA samples were also analyzed by HPSEC-CAD. Separation of HA standards of varying molecular weights is shown in Fig. 3a. Figure 3b, c are representative HPSEC chromatograms of HA supplements. The sample in Fig. 3b is a polymer predominantly composed of a single peak. The shape of the peak in HPSEC is often indicative of the homogeneity of the sample. The non-symmetrical peak shape in Fig. 3b indicates the non-homogeneous nature of the sample. Figure 3c contains two non-symmetrical peaks indicating a heterogeneous sample. The difference in molecular size and purity can have significant impact in terms of biological efficacy. HPSEC-CAD offers an easy way to check for homogeneity and size of polysaccharides.

### Conclusion

This study demonstrated the utility of HPLC-CAD as a multipurpose analytical tool for QC analysis of carbohydrates. A HILIC-CAD method was developed for separation and detection of common uronic acids, amino sugars and select neutral sugars without derivatization. This method is an extension of other HILIC-CAD methods [9] for neutral sugar analysis. The HPLC-CAD platform can serve as a cheaper and simpler alternative to existing methods for composition analysis. Furthermore, the HPLC-CAD can be used to perform HPSEC experiments to analyze homogeneity and molecular weight of carbohydrate polymers.

## Limitations

The platform can only be used as a QC tool for known carbohydrate samples. Analysis of unknown samples can be difficult. Since CAD is a semi-universal detector, unwanted impurities owing to hydrolysis conditions might interfere with composition analysis of unknown samples. Also, the HILIC-CAD method proposed in this study cannot separate all neutral sugar residues such as glucose and galactose. Our method, which separates most common acidic and amino sugars, must be used in complementation with other reported neutral sugar separation methods for complete composition analysis.

## Additional file

**Additional file 1: Table S1.** Linearity, LOD, LOQ and precision of the proposed HILIC-CAD method. **Figure S1.** Effects of column temperature on chromatographic separation. **Figure S2.** Effects of tertiary amine TEA on chromatographic separation. **Figure S3.** Effects of ammonium acetate on chromatographic separation.

## Data Availability

Data will be available upon request.

## Abbreviations

QC: quality control
GC–MS: gas chromatography–mass spectrometry
HPLC: high performance liquid chromatography
HPAEC-PAD: high performance anion exchange chromatography-pulsed amphoteric detection
HILIC: hydrophilic interaction liquid chromatography
ELSD: evaporative light scattering detector
MS: mass spectrometer
RID: refractive index detector
CAD: charged aerosol detector
SEC: size exclusion chromatography
GlcN: d-glucosamine
GalN: d-galactosamine
GlcNAc: *N*-acetyl-d-glucosamine
GlcA: d-glucuronic acid
GalA: d-galacturonic acid
Neu5Ac: *N*-acetyl-neuraminic acid
LIdoA: l-iduronic acid
LFuc: l-fucose
Gal: d-galactose
Man: d-mannose
LRha: l-rhamnose
HA: hyaluronic acid
TFA: trifluoroacetic acid
TEA: triethylamine
LOD: limit of detection
LOQ: limit of quantification
RSD: relative standard deviation
HPSEC: high performance size exclusion chromatography
GPC: gel permeation chromatography
