# Supplementary material for: HPLC with charged aerosol detector (CAD) as a quality control platform for analysis of carbohydrate polymers
Source: BMC Res Notes. 2019 May 14;12:268. doi: 10.1186/s13104-019-4296-y (PMC6518655; doi:10.1186/s13104-019-4296-y)
Supplement: Supplementary file 1 — Additional file 1: Table S1. Linearity, LOD, LOQ and precision of the proposed HILIC-CAD method. Figure S1. Effects of column temperature on chromatographic separation. Figure S2. Effects of tertiary amine TEA on chromatographic separation. Figure S3. Effects of ammonium acetate on chromatographic separation. [file 13104_2019_4296_MOESM1_ESM.docx]

| Monosaccharide | Regression equation | R^2^ | LOD (ng/mL) | LOQ (ng/mL) | Precision (Peak Area) | |
| --- | --- | --- | --- | --- | --- | --- |
|  |  |  |  |  | Intra-day (%RSD) | Inter-day (%RSD) |
| LRha | y = 0.7226x – 1.3072 | 0.9965 | 68.82 | 232.35 | 3.99 | 2.49 |
| LFuc | y = 0.8565x – 1.5429 | 0.9910 | 65.45 | 218.18 | 1.32 | 2.66 |
| Man | y = 0.8879x – 1.8010 | 0.9993 | 50.51 | 169.09 | 4.86 | 2.01 |
| Gal | y = 0.8975x – 1.7640 | 0.9946 | 55.42 | 184.75 | 2.50 | 0.92 |
| GlcN | y = 0.9491x – 2.1228 | 0.9986 | 64.77 | 215.91 | 1.93 | 3.16 |
| GalN | y = 0.9599x – 2.3001 | 0.9952 | 61.71 | 205.71 | 3.07 | 1.76 |
| GlcNac | y = 0.8439x – 1.7494 | 0.9940 | 64.05 | 213.51 | 4.67 | 4.70 |
| GluA | y = 0.7994x – 1.5368 | 0.9954 | 63.00 | 210.00 | 7.96 | 2.64 |
| GalA | y = 0.9350x – 1.8938 | 0.9943 | 64.08 | 213.61 | 4.16 | 7.17 |
| IdoA | y = 0.9070x – 1.8958 | 0.9979 | 83.40 | 278.00 | 5.96 | 4.61 |
| Neu5Ac | y = 0.8129x – 1.4928 | 0.9977 | 53.08 | 176.92 | 5.14 | 2.43 |

**Additional figures and tables**

**Table S1:** Linearity, LOD, LOQ and precision of the proposed HILIC-CAD method.


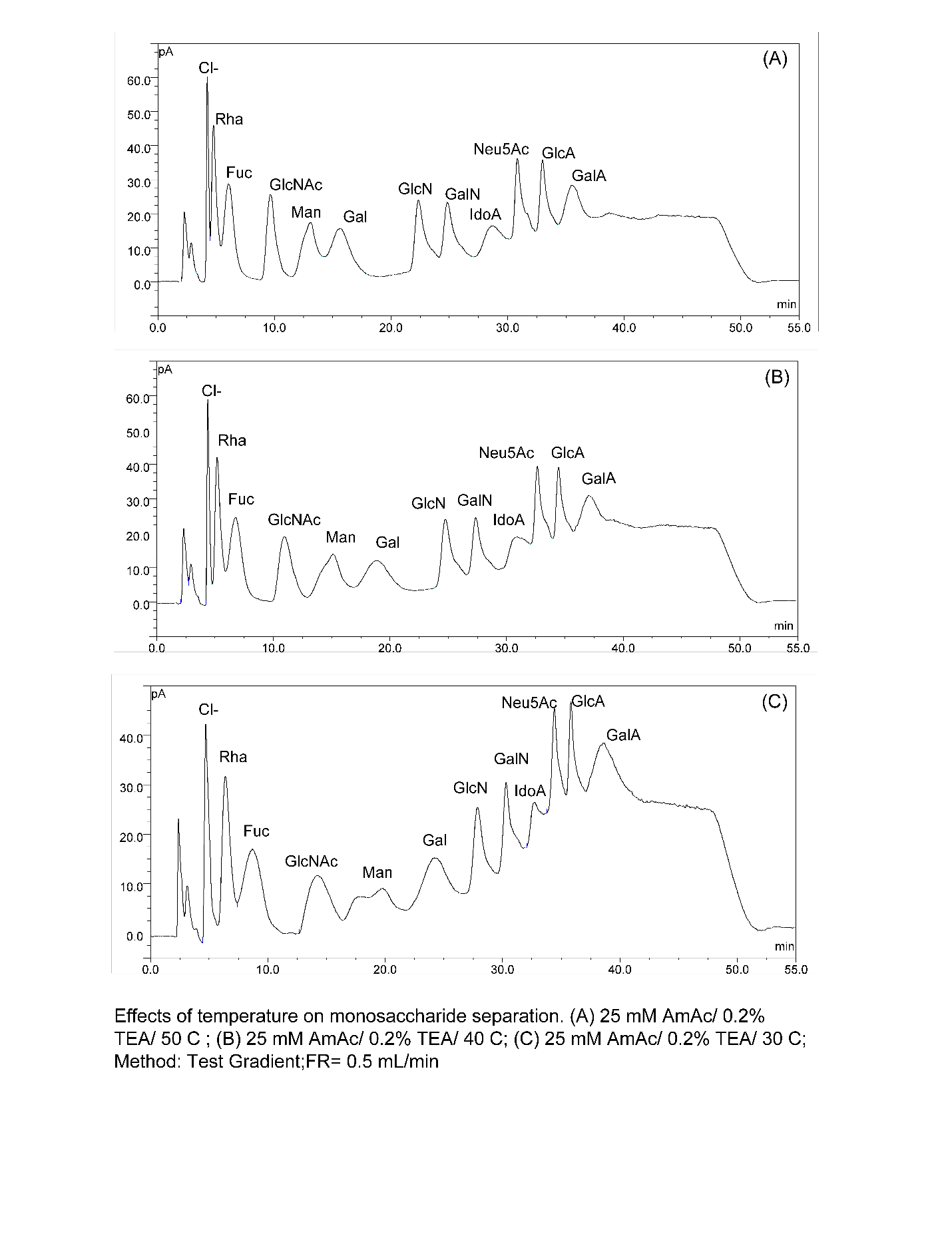


**Figure S1. Effects of column temperature on chromatographic separation.** Mixtures of 11 monosaccharide standards (concentration: 225 µg/mL) were analyzed in A-C. Mobile phase additives and column temperature in: (A) 25 mM ammonium acetate/0.2% TEA/50°C; (B) 25 mM ammonium acetate/0.2% TEA/40°C; and (C) 25 mM ammonium acetate/0.2% TEA/30°C. The mobile phase gradient reported in the methods section was used for the separation. Although, lower temperatures lead to better separation of monosaccharides, they tend to form peak doublets below 30°C. An example of peak doublet (Man) can be seen in panel C. Higher temperatures of >30°C were found to adequately suppress doublets.


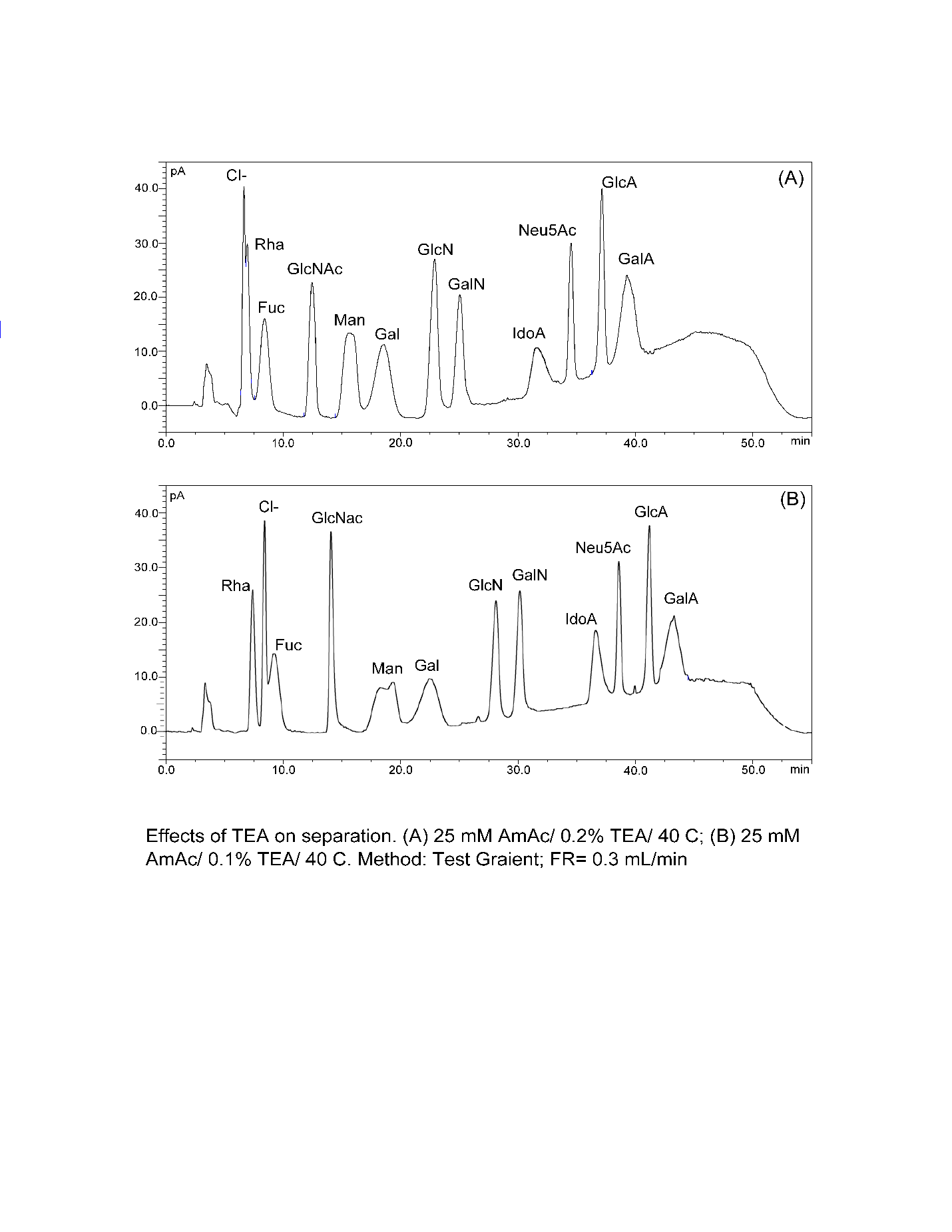


**Figure S2. Effects of tertiary amine TEA on chromatographic separation.** Mixtures of 11 monosaccharide standards (concentration: 225 µg/mL) were analyzed in A and B. Mobile phase additives and column temperature in: (A) 25 mM ammonium acetate/0.2% TEA/40°C; and (B) 25 mM ammonium acetate/0.1% TEA/40°C. The mobile phase gradient reported in the methods section with a flow rate of 0.3 mL/min was used for the analysis. A combination of 0.2% TEA and temperatures above 30°C were found to suppress split peaks.

**
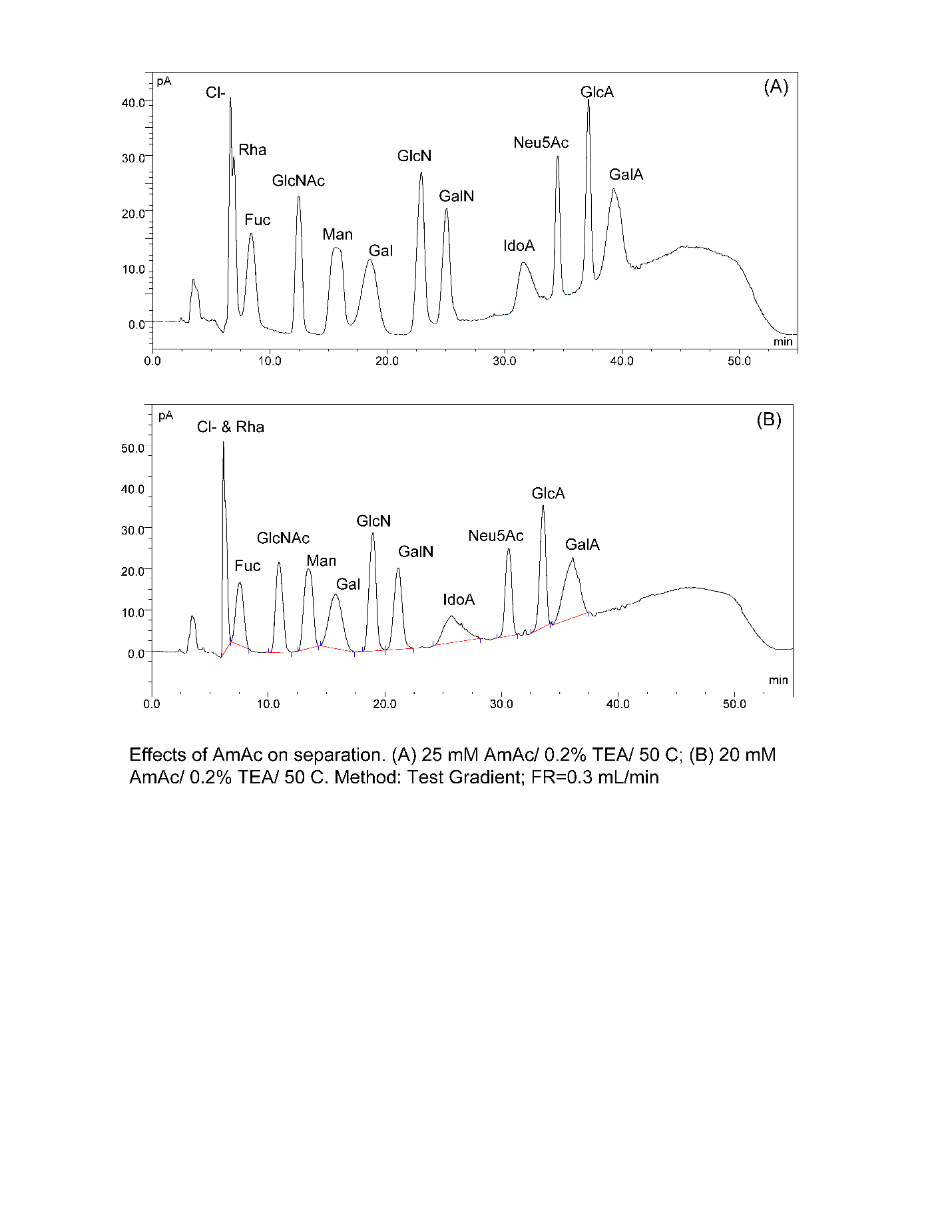
**

**Figure S3. Effects of ammonium acetate on chromatographic separation.** Mixtures of 11 monosaccharide standards (concentration: 225 µg/mL) were analyzed in A and B. Mobile phase additives and column temperature in: (A) 25 mM ammonium acetate/0.2% TEA/50°C; and (B) 20 mM ammonium acetate/0.2% TEA/50°C. The mobile phase gradient reported in the methods section with a flow rate of 0.3 mL/min was used for the analysis. Although, higher ammonium acetate buffer concentrations lead to increased retention times, it was generally found to produce better separations.
